# Overall Quality of Sporting Events and Emotions as Predictors of Future Intentions of Duathlon Participants

**DOI:** 10.3389/fpsyg.2020.01432

**Published:** 2020-07-17

**Authors:** Ana Mᵃ Magaz-González, César Sahelices-Pinto, Cristina Mendaña-Cuervo, Marta García-Tascón

**Affiliations:** ^1^Departament of Didactic of Musical, Plastic and Corporal Expression, Faculty of Education, University of Valladolid, Soria, Spain; ^2^Departament of Economic and Business Administration, Faculty of Economics and Business Administration, University of Leon, Leon, Spain; ^3^Department of Sports and IT, Faculty of Sport Sciences, Pablo de Olavide University, Seville, Spain

**Keywords:** future intentions, general satisfaction, perceived value, emotions, sport events participants, structural equations modeling, fuzzy set qualitative comparative analysis

## Abstract

The present study is intended to analyze the effect of global quality, perceived value, general satisfaction, and emotions on future behavior patterns among participants in the European Duathlon Championship. In this sense, a questionnaire was administered (*n* = 210), composed of four sections: essential demographic and profiling variables, perceived quality, overall quality, and emotions. Consequently, a relational model was designed to be examined by means of structural equation modeling (SEM) and fuzzy set qualitative comparative analysis (fsQCA). Results reveal that global quality and general satisfaction are key dimensions for determining future behaviors of participants, but not so the perceived value. Moreover, up to three combinations of these dimensions together with emotions -pleasure and arousal- emerged as enough for depicting future intentions to a great extent. In this line, a remarkably sufficient combination consists of global quality, general satisfaction, pleasure, and arousal. These findings will guide organizers to design strategies that provide exciting experiences, as well as quality and satisfaction to the participants of sports events.

## Introduction

Sporting events have become a major economic activity (Alonso-Dos-Santos, 2018; Lin and Lu, 2018; Zhang et al., 2018; Almeida, 2019) and sports companies and organizations in charge of their organization are subject to competitive forces like other markets, and so must seek strategies to differentiate themselves. The proliferation of this type of event results in athletes having to choose between multiple offers in the annual calendar and are facing increasingly demanding choices. The quality of the event that the participant perceives, the value that it gives to them, and the satisfaction that it causes are key elements for athletes’ loyalty to the sporting event and their future intention of returning to that sporting event. Furthermore, the emotions experienced with their participation in the event shape their behavior (Calabuig-Moreno et al., 2015) and can influence their choice. On their part, organizations are interested in customer loyalty. So, for the organizing committee it is important to identify the quality perceived, the satisfaction, the value, the future intention, and the emotions experienced by participants, and to understand how they impact each other and manage them, in order to be more competitive.

In the service literature, understanding the relationship between quality, perceived value, satisfaction, and customer loyalty is a topic that has been analyzed in depth (Parasuraman et al., 1985, 1988; Zeithaml et al., 1996). Sports services are the focus of many of these analyses within the field of sports management (Cronin et al., 2000; Hightower et al., 2002; Calabuig-Moreno et al., 2010; Ko et al., 2010; Theodorakis et al., 2013; Alguacil et al., 2019). Few, however, add to the study the role of emotions (Biscaia et al., 2012; Calabuig-Moreno et al., 2015; Crespo-Hervás et al., 2019), despite the fact that in sports services, emotions and feelings are important factors for participation and development (Pérez-Campos, 2010; Vacher et al., 2017; Crespo-Hervás et al., 2019). Most of the literature is focused on viewer satisfaction, but there is less research focused on participants. Therefore, this study investigates a model that explains the relationships between service quality, perceived value, satisfaction, and emotions, and their ability to predict the future intentions of participants in a European Duathlon championship. The added value of this research lies, firstly, in introducing the role of emotions as a moderating variable, secondly, in applying it in a specific sporting event, and finally, in analyzing the relationships with two complementary methodologies to compare their results and reinforce or not the proposed model: structural equation modeling (SEM) and qualitative analysis (fsQCA).

## Conceptual Framework and Hypothesis

The success of a sporting event depends on the degree to which it satisfies the participants and spectators with a quality service (Ko et al., 2010). Quality perception is formed through an individual’s opinion of the superiority of a service derived from comparing consumption expectations and perceptions of the real performance of the service. It involves evaluating service delivery (Parasuraman et al., 1985) and the outcome (Brady and Cronin, 2001; Ko et al., 2011). Perceived quality is a multidimensional and hierarchical construct. The SERVQUAL instrument (Parasuraman et al., 1985) explains it using five dimensions. Others do so through dimensions and sub-dimensions (Brady and Cronin, 2001; Clemes et al., 2011; Ko et al., 2011; Yoshida and James, 2011). In the area of sporting events, studies focus on the quality perception of service users or spectators of sporting events. Instruments such as SPORTSERV (Theodorakis et al., 2001), EVENQUAL (Calabuig-Moreno and Crespo-Hervás, 2009), SEQSS (Ko et al., 2011), EPOD, and EPOD2 (Nuviala et al., 2008, 2013) are commonly used. The structure of these models is affected by the characteristics of each sporting event or service, adding specific dimensions to the particular context (Theodorakis et al., 2015; Calabuig-Moreno et al., 2016b; Choi et al., 2018; García-Fernández et al., 2018). But at a sporting event, participants may perceive quality differently than spectators (Shonk and Chelladurai, 2008). Taking this premise into account, several investigations guide their models to analyze the quality perceived by the participants (Ko et al., 2010; Pérez-Campos, 2010; Chen et al., 2012; Angosto-Sánchez et al., 2016a, b; Montesinos-Saura et al., 2018). Angosto-Sánchez (2014) includes the perceived quality assessment instruments and the dimensions proposed by these authors in the area of sports services. Martínez-García and Martínez-Caro (2010) emphasize that clients can form perceptions of service quality at different levels of abstraction, from the most aggregated (such as overall service quality) to the most disaggregated, with different quality sub-dimensions. They propose the inclusion of a measure of overall service quality. With this, it is possible to verify if the global evaluation of service quality is different from the evaluation of the diverse attributes of perceived quality, to analyze the degree of solidity of the overall evaluation of service quality, check if some attributes are poorly rated, and compare them with the client’s affective judgment. Along this same line, Clemes et al. (2011) reflect that the perceptions of different main dimensions form a global perception of the quality of the service and these, in turn, influence the perceived value and satisfaction of a service. Pérez-Campos (2010) and Angosto-Sánchez (2014), based on the instrument of Hightower et al. (2002), introduce the evaluation of global service quality in their questionnaires.

H1: There is a direct and positive relationship between the different dimensions of quality perceived by the participant and the global quality of the event. This hypothesis is broken down into the following:

H1a: The good treatment of the organization’s staff positively influences the global quality perception of the event.

H1b: The effective communication of the event positively influences the global quality perception of the event.

H1c: Good complementary services positively influence the global quality perception of the event.

H1d: Effective logistics positively influence the perception of the global quality of the event.

H1e: Correct management of the specific elements of the duathlon positively influences the perception of the global quality of the event.

For service managers, including those at sporting events, it is also important to know the perceived value of the service by customers as it is a predictor of behavioral intentions and loyalty and allows for less sensitivity toward price. The perceived value results from evaluating the utility of a product or service by comparing what is received with what is given (Zeithaml, 1988). Thus, consumers perceive value when the organization personally satisfies them in relation to their tastes, preferences, or needs and in comparison, with what they have invested in time, money, effort, and sacrifice (Calabuig-Moreno et al., 2010; Boksberger and Melsen, 2011). Quality is identified by several authors as a predictor of perceived value and as an antecedent of future intentions (Cronin et al., 2000; Hightower et al., 2002; Murray and Howat, 2002; Nuviala et al., 2015; García-Fernández et al., 2018).

H2: There is a direct and positive relationship between global quality and perceived value.

The global quality of service and the dimensions it describe are directly related to satisfaction (Rust and Oliver, 1994; Zeithaml et al., 1996; Brady and Cronin, 2001), to the extent that the second factor is considered a consequence of the previous. Satisfaction is understood to depend on the discrepancy between expectations before consumption and perceptions of the service consumed (Oliver, 1980; Shonk and Chelladurai, 2008) and is also transitory, associated with a specific situation (Parasuraman et al., 1988). In sports services, where there is a high emotional involvement, the emotional attachment conditions these (Westerbeek and Shilbury, 2003; Alonso-Dos-Santos and Pérez-Campos, 2015). The participant satisfaction is thus an attitudinal result after the race participation. However, even the pre-competition motivational aspects can condition an individual to make the decision to register and compete (Du et al., 2015). Therefore, this decision is influenced by experience, subjective perception of service (Westerbeek and Shilbury, 2003; Montesinos-Saura et al., 2018; Bi et al., 2019), and by cognitive and affective elements (Oliver, 1981). In this line, satisfaction is linked to behavioral future intentions and loyalty (Alonso-Dos-Santos and Pérez-Campos, 2015; Bernal-García et al., 2018; Alguacil et al., 2019) and creates less price-sensitive customers (Ko et al., 2010; Calabuig-Moreno et al., 2015; Montesinos-Saura et al., 2018). Matsuoka et al. (2003) suggest that satisfaction has a strong effect on future intentions and the decision to attend other events. For its part, Biscaia et al. (2012) also identify a significant relationship between “satisfaction” and “behavioral intentions” so that satisfied spectators are more likely to attend future games, recommend them to others, and purchase equipment products and services. These results are consistent with several previous studies in sports settings (Matsuoka et al., 2003; Kuenzel and Yassim, 2007; Yoshida and James, 2010). Theodorakis et al. (2015) study how different dimensions of quality impact on the satisfaction of participants in sporting events. Nuviala et al. (2013) carry out an adaptation of previous questionnaires (Oliver, 1980, 1997; Bodet, 2006) in their EPOD2 instrument to assess satisfaction in sports services. And Pérez-Campos (2010) and Angosto-Sánchez (2014) use an adaptation of the scale of Hightower et al. (2002) to measure it in participants of sporting events. All of them consider emotional aspects to describe satisfaction.

H3: There is a direct and positive relationship between global quality and general satisfaction.

Another issue for managers to consider is the relationship between perceived value and satisfaction. There is controversy surrounding this issue in the literature. The works establish both a direct and an inverse, non-existent, and even reciprocal relationship between both dimensions (Nuviala et al., 2013, 2015, 2020; Bernal-García et al., 2018). But in service companies, including sports services companies, the majority defend that the perceived value affects satisfaction (McDougall and Levesque, 2000; Hightower et al., 2002; Murray and Howat, 2002; Brady et al., 2005; Calabuig-Moreno et al., 2015; García-Fernández et al., 2016; Crespo-Hervás et al., 2019).

H4: There is a direct and positive relationship between perceived value and general satisfaction.

For event organizers, knowing how to influence the participant’s behavior to repeat the experience is a challenge. Following Biscaia et al. (2012), the behavioral intention is the participant’s favorable intention to attend future games or recommend them to others, as well as purchase products associated with the event. Zeithaml et al. (1996) argue that behavioral intentions diagnose actual behaviors better than quality and satisfaction (Clemes et al., 2011). On the other hand, there are multiple studies that point to satisfaction and perceived value as antecedents of future intentions, and, therefore, indirectly to global quality, as a precursor to these (Zeithaml et al., 1996; McDougall and Levesque, 2000; Murray and Howat, 2002; Brady et al., 2005). So, knowing how these relationships develop and what motivates the intention to participate in an event again is important for managers. This statement is also shared in the sports context (Cronin et al., 2000; Hightower et al., 2002; Matsuoka et al., 2003; Pérez-Campos, 2010; Clemes et al., 2011; Theodorakis et al., 2013; Calabuig-Moreno et al., 2014). Many authors include in their models the simultaneous analysis of the effects – whether direct or indirect – of quality, satisfaction, and value in future intentions.

H5: There is a direct and positive relationship between perceived value and future intentions.

H6: There is a direct and positive relationship between general satisfaction and future intentions.

In the related service literature, a common assumption points that future intentions may be derived from other factors, such as the emotions that arise in a sporting event (Sumino and Harada, 2004; Biscaia et al., 2012). Emotions are complex interactions between subjective and objective factors influenced by neuronal and hormonal systems that generate feelings, cognitive processes, and activation of physiological functions and behaviors (Kleinginna and Kleinginna, 1981; Biscaia et al., 2012). These types of events are characterized by a high level of emotional participation (Mullin et al., 2007) and by generating hedonic experiences and varied moods in the participants. Thus, passion is the origin of high participation (Crespo-Hervás et al., 2019), and the sense of personal efficacy for achieving performance provokes a positive emotional affinity with the event (Du et al., 2015). Therefore, these emotions can create stronger emotional bonds than cognitive judgments (Fournier, 1998) and affect the perception of service performance (Calabuig-Moreno et al., 2015) such as satisfaction, perceived value, and future commitment to the service (Sumino and Harada, 2004; Biscaia et al., 2012). For instance, Oliver (1997) suggests that satisfaction depending on experience involves emotions, and Biscaia et al. (2012) suggests that a behavioral intention is better predicted if measures of satisfaction and emotion are used. Although these intangible variables are difficult for managers to manage, it is interesting to analyze the moderating effect of these on the evaluation of the service and on future intentions. However, there are few studies that incorporate emotions in the analysis in sporting events (Sumino and Harada, 2004; Pérez-Campos, 2010; Biscaia et al., 2012; Alonso-Dos-Santos and Pérez-Campos, 2015; Calabuig-Moreno et al., 2015, 2016a,b), and these reach different conclusions. In this case, we evaluate how the emotions of the participants in the sporting event moderate the effect of perceived value and general satisfaction on athlete’s intention to participate in future sporting events. For this, the pleasure-arousal taxonomy is used (Russell, 1980), as suggested by other authors, as it is a stable model in leisure contexts (Calabuig-Moreno et al., 2015, 2016a). Russell (1980) evidences that the affect dimensions are interrelated in a systematic fashion and it is possible to explain them with a circumplex model of affect. Russell (1980) represents this interrelation by a spatial model in which affective concepts fall in a circle in the following order: pleasure (0°), excitement (45°), arousal (90°), distress (135°), displeasure (180°), depression (225°), sleepiness (270°), and relaxation (315°). In this way, Russell’s taxonomy very comprehensively captures affective experience and allows any word of affect to be defined as a combination of the components of pleasure and arousal.

H7: The inclusion of pleasure-arousal emotions in the model improves its explanatory power.

H8: Pleasure positively moderates the effect on future intention. It needs to derive two sub-hypotheses from the initial one:

H8a: Pleasure positively moderates the effect of perceived value on future intention.

H8b: Pleasure positively moderates the effect of general satisfaction on future intention.

H9: Arousal positively moderates the effect on future intention. It needs to derive two sub-hypotheses from the initial one:

H9a: Arousal positively moderates the effect of perceived value on future intention.

H9b: Arousal positively moderates the effect of general satisfaction on future intention.

**FIGURE 1:**
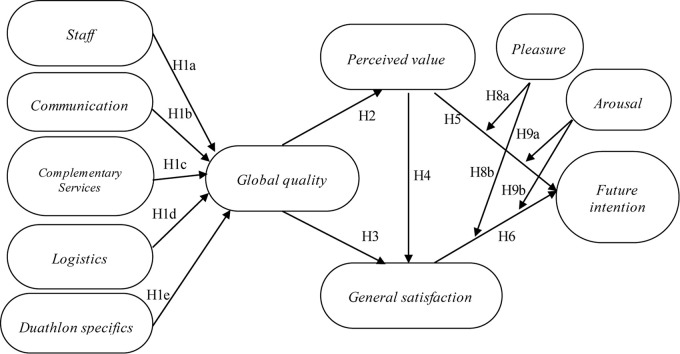
The research structural model. Global quality constructs, perceived value, general satisfaction and emotions, and the effect of athlete’s intention to participate in future sporting events.

In summary, the research model tests the effects of the following constructs: the dimensions of perceived quality on the global quality of service, the global quality of service on perceived value, and general satisfaction; the perceived value in general satisfaction and future intentions; and general satisfaction in future intentions. It also measures the effect of pleasure and arousal emotions on future intentions through its moderating effect on value and general satisfaction. Observed in Figure 1.

## Materials and Methods

### Participants

In order to address the investigation hypotheses discussed above, a research approach was conducted based on a survey administered to a final sample of 210 participants (*N* = 999; *e* = ± 6.13%; α = 95.5) in the European Duathlon Championship, celebrated in Soria (Spain) in 2017. The duathlon is an individual sport that can be regarded as a modality of a triathlon. The sample comprised 151 males (71.9%) and 59 females (28.1%), aged 17–75 years old (μ = 41.16; σ = 14.22). In this championship, there may be professional participants who are scoring within the general ranking as well as amateur participants.

### Measures

All participants were asked to voluntarily answer a questionnaire consisting of four main sections (via computer aided personal – CAPI- and web -CAWI- interview). The first section gathered information about essential demographic and profiling variables. Likewise, the second section was integrated by diverse scales concerning the five components or sub-dimensions of perceived quality (staff –four items-, communication -four items-, complementary services -five items-, logistics -four items-, and specifics aspects of duathlon -eight items). The third section was devoted to the measurement of the overall quality of the event. This was a multidimensional instrument divided into four concepts, namely, global quality –four items-, perceived value –four items-, general satisfaction –four items-, and future intentions –five items-. All these scales were adapted from the previous research of Angosto-Sánchez et al. (2016b). The fourth section assessed the diverse emotions linked to the performance of individuals at the event. It was composed of 10 emotions referring to the basic spheres of pleasure (five items) and arousal (five items), originally developed by Russell (1980) and replicated by Pérez-Campos (2010) or Calabuig-Moreno et al. (2016a). All responses took the mode of a five-point Likert scale ranging from 1 (strongly disagree) to 5 (strongly agree).

Following this line, a group of experts was selected in order to assess the content validity of the instrument (Skjong and Wentworth, 2001; Hernández-Sampier et al., 2010; Muñiz and Fonseca-Pedrero, 2019). These experts were both academics and professionals in the sports industry and presented suggestions about the potential deletion/modification of existing items, and/or the inclusion of prospective ones. Particularly, the criterion considered to add an item to the final version of the instrument required the agreement of at least 80% of the experts (Hyrkäs et al., 2003).

In the assessment, the group of experts was composed of 11 gender-equal participants: three male university professors, with extensive experience (more than 10 years) and experts on the subject matter, from different Faculties of Sports Sciences of Spanish universities; four athletes, with more than 6 years of competition experience (two female professionals and two male amateurs); and four members of the organization with more than 15 years of experience (two females belonging to the City hall of the championship host city and two men from the organization of the event).

The group of experts analyzed the content of the different items and their relevance, clarity, simplicity, and comprehensibility in relation to the object of study (Thomas and Nelson, 2007). After this phase, five items were eliminated from the sociodemographic section: three related to the perceived quality and one to the overall quality. In addition, four items referring to the specific duathlon test were added. Eventually, the wording of two items of the staff dimension were modified for improved comprehension.

### Data Analysis

After the qualitative validation, the factorial unidimensional, convergent, and discriminant validities were statistically assessed.

Primarily, with the intent to test the proposed model, a structural equation modeling (SEM) technique of PLS was used, with the software SmartPLS 2.0 (Ringle et al., 2005). In contrast to other methods, such as covariance-based ones which are focused on the estimation of model parameters and overall fit measures, the objective of PLS is to maximize the variance explained by indicators and latent variables through the estimation of ordinary least squares and principal components analysis. In this frame, data treatment responds to the creation of optimal linear predictive paths with minimal demands on measurement scales, residual distributions, and sample sizes (Fornell and Bookstein, 1982; Chin, 1998). Therefore, compared with maximum likelihood methods, the PLS approach better matches the requirements of exploratory and theory building applications (Barclay et al., 1995; Chin et al., 2003).

The second stage, aimed at supporting and ratifying the previous analysis, the methodology fsQCA (Ragin, 2008) was applied in order to reach a more holistic and precise identity about those antecedents and consequences that represent outer or explanatory variables and research findings in case studies like the present investigation. Indeed, this methodological approach is centered on the estimation not of independent net effects but of combinatory ones, so that it is intended to identify all possible conditions –both necessary and sufficient- that lead to a specific result.

Overall, fsQCA (Ragin, 2008) is an approach that examines sets of established relationships emerged from the conjunction of the Qualitative Comparative Analysis –QCA- (Ragin, 1987), and the theory of fuzzy sets posed by Zadeh (1965). In relation to the QCA, the procedure starts from all possible combinations between observed variables to determine what implications data support through the application of techniques of logic inference. Likewise, in the traditional theory of sets, membership is defined in binary terms, that is, an element belongs –value 1- or does not belong–value 0-to a set. However, in fuzzy sets, the same element is allowed to belong to a set in a certain degree: value 1 is linked to those elements that belong to the set without any sort of doubt, and value 0 to those that do not, whereas intermediate values are associated to elements with questionable membership, adopting diverse degrees of belonging in the range 0.0–1.0. This fact indicates that the same element may belong to different sets at a time in different degrees of membership. In this sense, the absence of strict limits between sets endows flexibility to the decision-making. For this stage, the software fsQCA 3.0^1^ was used.

In particular, with regard to the sample size reached in this study, it is worth mentioning that both methodological approaches, PLS and fsQCA, widely used in the area of management and organizational issues, are particularly appropriate for operating in those research situations where sample sizes are limited (Chin and Newsted, 1999; Ragin, 2008; Rihoux and Ragin, 2009; Hair et al., 2012) and databases are derived from surveys (Emmenegger et al., 2014).

## Results

### Assessment of the Measurement Model

#### Reliability of Instruments

Table 1 shows the items included in the measurement model and their psychometric properties for the full sample. With the intent of evaluating the internal consistency of scales, the PLS technique produces three indicators: Cronbach’s alpha (α) (Nunnally and Bernstein, 1994), composite reliability (ρ_*c*_), and average variance extracted (AVE) indexes (Fornell and Larcker, 1981). Referring to α and ρ_*c*_, the latter is considered by some authors to be superior to the former because it is independent of the number of attributes associated with each construct (Fornell and Larcker, 1981). The interpretation of both indices is similar and values above 0.70 are considered reasonable (Bagozzi and Yi, 1988; Nunnally and Bernstein, 1994; Barclay et al., 1995). The results obtained showed compliance with this requirement, ensuring minimized measurement error (communication scale reflects the lowest coefficient rising to 0.73). For its part, AVE indexes, which quantify the amount of variance that a construct captures from its indicators relative to the amount of variance due to measurement error (Chin, 1998), were obtained through the execution of confirmatory factorial analysis (CFA). AVE values appeared to be satisfactory since these took positions above the minimum benchmark of 0.50 for all latent variables (Hair et al., 2006), as can be checked in Table 1 (0.90 for S, 0.79 for C, 0.80 for CS, 0.77 for L, 0.70 for DS, 0.90 for GQ, 0.85 for PV, 0.92 for GS, 0.80 FI, 0.81 for P, and 0.87 for A).

**TABLE 1:** Psychometric properties of scales.

Constructs and items^*a*^	λ	t	λ^2^	α	ρ_*c*_	AVE
**Staff (S)**				0.8837	0.9450	0.8958
The organizing committee meets scheduled timetables	0.9481***	64.456	0.8989			
The staff are ready to help and give advice	0.9448***	50.7746	0.8926			
**Communication (C)**				0.7283	0.8803	0.7862
The event comes with a fair promotion and diffusion, and provides enough practical information about it	0.8935***	39.9111	0.7983			
It was easy to register as a participant	0.8799***	23.0448	0.7742			
**Complementary Services (CS)**				0.7536	0.8895	0.8010
The event has enough support utilities (WC, showers, changing rooms, cloakroom, massage zone, stands, etc.)	0.8745***	27.9938	0.7648			
There are easily accessible places (cafeterias, bars, restaurants.) close to the start/finish line	0.9151***	48.1548	0.8374			
**Logistics (L)**				0.8888	0.9363	0.7665
Event signs facilitate reaching the start line easily	0.8906***	19.4209	0.7295			
Material elements used at the event are visually appealing (banners, billboards, start line, finish line, circuit…)	0.9061***	23.8639	0.7661			
**Duathlon Specific (DS)**				0.8538	0.9013	0.6956
The equipment check has been carried out easily and without excessive waiting time	0.8160***	16.4028	0.6659			
The race comes with the necessary security measures to ensure the proper surveillance of the equipment	0.8739***	30.7506	0.7637			
The transition point is spacious and tidy to allow for adequate flow without clustering	0.8131***	15.5884	0.6611			
The circuit ground is in satisfactory conditions, with turns properly signed, and is free of obstacles	0.8316***	20.4228	0.6916			
**Global Quality (GQ)**				0.8946	0.9499	0.9046
Overall, the service provided by the organizing committee is appropriate	0.9529***	80.6094	0.9080			
I consider that the involvement of the staff in the event has been excellent	0.9493***	70.6986	0.9012			
**Perceived Value (PV)**				0.8272	0.9201	0.8520
I think that the event has been reasonably priced in general	0.9110***	9.8179	0.8299			
Overall, I consider that the race has a good quality-price relation	0.9349*	1.7696	0.8740			
**General Satisfaction (GS)**				0.9172	0.9602	0.9234
I am glad about all experiences I have had in this event	0.9644***	97.8323	0.9301			
Indeed, I have enjoyed participating in this event	0.9575***	61.4438	0.9168			
**Future Intentions (FI)**				0.9136	0.9405	0.7994
I stand ready to continue participating in future editions of the race	0.9465***	63.7668	0.8959			
I will recommend other athletes, friends, relatives, other people. to participate in the championship	0.9326***	47.6816	0.8697			
If I have the opportunity to participate in a similar event, I will repeat that experience	0.9306***	45.6017	0.8660			
**Pleasure (P)**				0.9461	0.9564	0.8144
Glad	0.9322***	4.6063	0.9655			
Delighted	0.8403***	3.9967	0.9167			
Pleased	0.8827***	4.5995	0.9395			
Excited	0.9357***	4.9239	0.9673			
Happy	0.9179***	3.5431	0.9581			
**Arousal (A)**				0.9630	0.9713	0.8715
Distressed	0.9093***	4.8802	0.9536			
Angry	0.9649***	5.3472	0.9823			
Annoyed	0.9522***	4.7832	0.9758			
Tense	0.8771***	4.4270	0.9365			
Afraid	0.9608***	5.2310	0.9802			

^*a*^λ, Factorial loading; λ^2^ Communality; α, Cronbach’s alpha; ρ_*c*_, Composite reliability; AVE, Average Variance Extracted. *p < 0.05; **p < 0.01; ***p < 0.001.

#### Validity of the Instruments

Subsequently, convergent and discriminant validities were checked for estimating the robustness of the scales. Commonly assumed, convergent validity is ascertained by verifying the significance of the standardized loadings (λ) in the CFA (Barclay et al., 1995; Chin, 1998), and that each one of the dimensions included in the study is significantly correlated with the rest (Gómez-Bernabeu and Palací, 2003). In this sense, all loadings were above the threshold 0.50 (Barclay et al., 1995; Chin, 1998), according to a significance level of 95% (*p* < 0.05) and calculated based on 200 bootstrapping runs, except for items 1 and 4 of S, 2 and 4 of C, 1, 2, and 3 of CS, 2 and 4 of L, 3, 5, 7, and 8 of DS, 2 and 4 of GQ, 3 and 4 of PV, 2 and 4 of GS, and 4 and 5 of FI, which did not reach this threshold and were consequently removed from the study (up 21 to items were excluded). Likewise, item communalities (λ^2^) exceeded the minimum requirement of 0.25, such that the latent constructs explained between 66.1 and 98.2% of variance in their respective observed indicators. Similarly, as seen in Table 2, the correlations between pairs of constructs were also significant, although timid correlations could be identified between L, PV, P, and A, and the rest of the dimensions of the model, which seems to anticipate the poor explanatory/predictive capacity of these constructs over the hypotheses proposed in previous sections in reference to FI. Apart from that, a reasonable convergent validity can be confirmed (Anderson and Gerbing, 1988).

**TABLE 2:** Latent variable correlation matrix and square roots of AVE^a^*.

Constructs	Mean	*SD*	1	2	3	4	5	6	7	8	9	10	11
1. S	4.279	0.987	(0.9465)^a^										
2. C	3.881	0.996	0.7506	(0.8867)									
3. CS	3.624	1.161	0.6539	0.6686	(0.8950)								
4. L	3.787	1.111	0.0231	0.0275	–0.0190	(0.8755)							
5. DS	3.830	0.993	0.7462	0.7164	0.7755	0.0442	(0.8340)						
6. GQ	3.976	1.046	0.7978	0.7234	0.7059	0.0659	0.8251	(0.9511)					
7. PV	2.995	1.259	–0.0024	–0.0394	–0.0508	–0.0065	–0.0265	–0.0289	(0.9230)				
8. GS	3.976	1.189	0.6410	0.6724	0.6572	0.0297	0.7371	0.8109	–0.0251	(0.9609)			
9. FI	3.823	1.120	0.6616	0.6805	0.6627	0.0410	0.7233	0.8171	0.0432	0.8275	(0.8941)		
10. P	4.000	0.873	0.0310	0.0436	–0.0252	–0.0226	–0.0186	0.0961	–0.0297	0.1211	0.0868	(0.9024)	
11. A	1.407	0.758	0.0038	–0.0002	–0.0549	–0.0228	–0.0360	0.0779	–0.0300	0.0878	0.0636	0.9385	(0.9335)

^*a*^Square roots of √AVE are in parentheses; AVE, Average Variance Extracted (Table 1); SD, Standard Deviation. *p < 0.05.

Next, with regard to the discriminant validity, it was verified that the manifest variables correlations (Table 2) were stronger with their associated latent variable than with any other latent variable (Barclay et al., 1995), and not overall excessively high (<0.85), ensuring the existence of discriminant validity (Kline, 2005), except for emotional dimensions P and A. As Table 2 displays, the square roots of the AVE values (diagonal elements) were larger than the standardized correlations among constructs (off-diagonal elements), suggesting an overall satisfactory discriminant validity (Fornell and Larcker, 1981), but not for P (√AVE = 0.90) and A (√AVE = 0.93), whose correlation was 0.94. This finding reveals that there is a vague conceptual differentiation among participants referring to the two emotional dimensions P and A.

#### Confirmatory Factor Model

In order to confirm the factor structure of the re-specified model, which is composed of 11 dimensions and 31 indicators, authors used SEM through the PLS methodology. Such a technique, in contrast to other covariance-based structural equation modeling procedures, does not initially offer other global goodness-of-fit (GoF) measures different to the coefficient of determination R^2^ and AVE.

Nevertheless, even though new GoF measures have been recently formulated as indicative of judgment of the overall model fit in PLS path models, there is no clear consensus about its adequacy for this sort of approach, whose real potential revolves around its great capability for prediction rather than confirmatory purposes (Henseler and Sarstedt, 2013; Henseler, 2018).

The essential criterion in relation to R^2^ obtained for each endogenous construct points that it should be higher than 0.10 (Falk and Miller, 1992). As seen in Table 3, all latent variables exceeded that minimum requirement by far, with the exception of PV, which was 0.00.

**TABLE 3:** Partial effects of the structural relations, standardized loading, and hypothesis testing.

Hypothesis	Relations (path coefficients)	β(t)	Test
H1a	S → GQ	0.3506*** (3.6268)	Supported
H1b	C → GQ	0.0987 (1.0394)	Not supp
H1c	CS → GQ	0.0764 (0.7919)	Not supp
H1d	L → GQ	0.0374 (0.3114)	Not supp
H1e	DS → GQ	0.4319*** (3.8638)	Supported
H2	GQ → PV	−0.0289 (0.1100)	Not supp
H3	GQ → GS	0.8109*** (19.2160)	Supported
H4	PV → GS	−0.0017 (0.0241)	Not supp
H5	PV → FI	0.0626 (0.5818)	Not supp
H6	GS → FI	0.8291*** (10.7249)	Supported
H8	P → FI	−0.0594 (0.0095)	Not supp
H9	A → FI	0.1377 (0.0073)	Not supp
	R^2^ GQ	0.7639	
	R^2^ PV	0.0008	
	R^2^ GS	0.6576	
	R^2^ FI	0.6925	

*p < 0.05; **p < 0.01; ***p < 0.001.

### Structural Model

**FIGURE 2:**
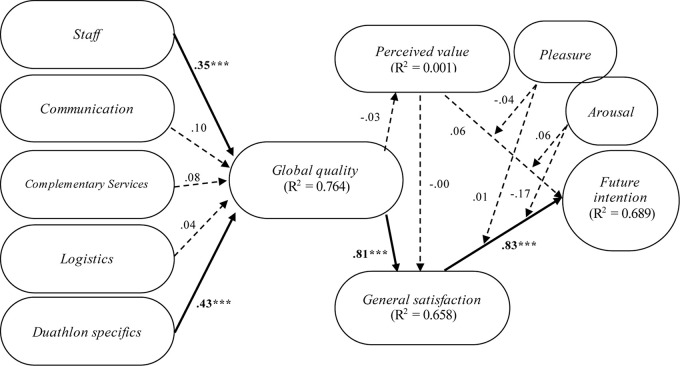
The structural model estimations. *p < 0.05; **p < 0.01; ***p < 0.001.

Once the reliability and validity of the measurement model were tested, PLS was used to assess the structural model (Figure 2). A bootstrapping procedure with 200 subsamples was applied to determine the statistical significance of each estimated path in the model, according to Student’s *t* computed for each hypothesis (Eberl, 2010).

Accordingly, in reference to hypotheses H1a and H1e, these were supported since statistical evidence that S (β_*S*_ → GQ = 0.35, *p* < 0.001) and DS (β_*DS*_ → GQ = 0.43, *p* < 0.001) have a positive impact over GQ (Table 3) was found. For their part, C, CS, and L did not seem to have any sort of effect on GQ. Hence, H1b, H1c, and H1d were not supported.

On the contrary, PV was confirmed to be a problematic construct since none of the research hypotheses in which it is implicated have emerged as significant (Table 3). That is, GQ did not appear to have any sort of effect on PV (H2 not significant), nor PV on GS (H4 not significant), nor PV on FI (H5 not significant). By contrast, a positive effect of GQ on GS (β_*GQ* → *GS*_ = 0.81, *p* < 0.001), and, in turn, GS on FI (β_*GS* → *FI*_ = 0.83, *p* < 0.001) came out as significant. H3 and H6 were thus supported (Table 3).

However, it should be noted that none of the two emotional dimensions, P and A, seemed to have any significant or enough of a positive moderating effect on the relations established in the model to explain FI of duathlon participants. Considering all output obtained, it would appear that the inclusion of emotions, measured in terms of P and A, does not provide greater explanatory nor predictive capacity to the model. Therefore, hypotheses H7, H8a, H8b, H9a, and H9b cannot be supported (Tables 3, 4).

**TABLE 4:** Overall effects.

Relations (path coefficients)	β (t)	Significance
S → PV	−0.0101 (0.1118)	No
S → GS	0.2843*** (3.5352)	Yes
S → FI	0.2351*** (3.2505)	Yes
C → PV	−0.0028 (0.0665)	No
C → GS	0.0800 (1.0414)	No
C → FI	0.0662 (1.0047)	No
CS → PV	−0.0022 (0.0486)	No
CS → GS	0.0620 (0.7951)	No
CS → FI	0.0512 (0.7686)	No
L → PV	−0.0011 (0.0157)	No
L → GS	0.0303 (0.3129)	No
L → FI	0.0251 (0.3014)	No
DS → PV	−0.0125 (0.1137)	No
DS → GS	0.3502*** (3.7268)	Yes
DS → FI	0.2896*** (3.4475)	Yes
GQ → FI	0.6705*** (10.1567)	Yes
PV * P → FI	−0.0389 (0.0155)	No
PV * A → FI	0.0634 (0.0023)	No
GS * P → FI	0.0124 (0.0256)	No
GS * A → FI	−0.1662 (0.0219)	No

*p < 0.05; **p < 0.01; ***p < 0.001.

Finally, the overall positive effect of S on GS (*ß*_*S* → *GS*_ = 0.28, *p* < 0.001), and on FI (ß_*S* → *FI*_ = 0.24, *p* < 0.001) emerged as statistically significant for the sample, as well as the positive effect of DS on GS (ß_*DS* → *GS*_ = 0.35, *p* < 0.001), and on FI (ß_*DS* → *FI*_ = 0.29, *p* < 0.001), and finally, the positive effect of GQ on FI (β_*GQ* → *FI*_ = 0.67, *p* < 0.001). No more overall positive effects between constructs were found to reach adequate levels of significance (Table 4).

In addition, Figure 2 and Table 3 also include indexes of global adjustment of the structural model. The essential criterion is the coefficient of determination (R^2^) obtained for each endogenous construct, which should be higher than 0.10 (Falk and Miller, 1992). All latent variables by far exceeded that minimum requirement, with the exception of PV, which was 0.00.

### Fuzzy-Set Qualitative Comparative Analysis (fsQCA)

Continuedly, bearing in mind all findings achieved through the previous approach, the next analysis is intended to verify whether emotions, if combined with other model dimensions, offer a more accurate explanation of FI. Furthermore, it is also deemed to be at the core of research objectives to know what combinations of conditions may elucidate the absence of FI to participate in sports events (∼FI).

Primarily, before implementing fsQCA, responses of participants must be transformed into fuzzy sets by the multiplication of the items scoring of latent variables (Villanueva et al., 2017; Crespo-Hervás et al., 2019). Thereupon, values of variables were calibrated, that is, it was determined to which extent each case belongs to each set. For the present study, since all figures range from 1 to 5, variables were directly calibrated considering percentiles 90, 50, and 10 as the basic thresholds (Woodside, 2013). Once calibration was carried out, those combinations that were not present among data in accordance with the table of configurations (truth table), or did not reach the minimum consistency cut-off and were conveniently deleted (due to the sample size, consistency cut-off was set in 0.90).

Referringto the necessary conditions test, all considered conditions reflected consistency values under the threshold 0.90 (Ragin, 2008) both for FI and for ∼FI, so it can be assumed that none of the variables represent a necessary condition of FI to participate or ∼FI to not participate. As usual, in the evaluation of sufficient conditions, the standard analysis presents three feasible scenarios: complex, parsimonious, and intermediate (the present study opts for the last one). Table 5 shows the referred output according to the notation employed by Fiss (2011).

**TABLE 5:** Sufficient conditions.

Frequency cut-off: 1	Future Intentions (FI)			∼Future Intentions (∼FI)
		
	Consistency cut-off: 0.93			Consistency cut-off: 0.91
		
	1	2	3	1
GQ	○	•	•	○
PV	•		•	○
GS		•	○	○
P	•	•	•	
A	•	•	•	•
Raw coverage	0.25	0.45	0.26	0.57
Unique coverage	0.02	0.21	0.07	0.57
Consistency	0.93	0.96	0.93	0.90
Overall solution coverage			0.55	0.57
Overall solution consistency			0.93	0.90

*^•^ Presence of condition. ○ absence of condition.*

Ragin (2008) and Woodside (2013) suggest that a solution is remarkable if it reflects a consistency score over the threshold of 0.74 and a coverage variation between 0.25 and 0.65. As shown in Table 5, all solutions meet both requirements. Concretely, there are three possible combinations of sufficient conditions (firstly ∼GQ^∗^PV^∗^P^∗^A, secondly GQ^∗^GS^∗^P^∗^A, and finally GQ^∗^PV^∗^∼GS^∗^P^∗^A) explaining up to 55% of FI, with a consistency of 0.93. On the other hand, a unique combination of sufficient conditions emerges as representative for ∼FI, which is ∼GQ^∗^∼PV^∗^∼GS^∗^A (solution coverage of 0.57; solution consistency of 0.90).

As seen before, the application of fsQCA allows analyzing interactions between different independent variables, an aspect that SEM does not permit. Overall, although it is clear that no necessary conditions exist, there are some possible combinations (sufficient combinations) that may stimulate FI to participate in sports events. In this sense, it can be assumed according to results that emotions play a relevant role at the time of influencing FI, since both dimensions (P and A) are present in all combinations of solutions extracted. Therefore, hypothesis H7 can be supported. Instead, in case of ∼FI, findings support that high rates of A are countered by the absence of the other explanatory variables of the model (GQ, PV, and GS).

## Discussion and Conclusion

For several years, participation in sporting events has been booming and has become a form of both leisure and competition, attracting great social, political, and economic interest. The organization of sporting events is a challenge for federations, companies, and cities that strive to develop strategies to improve the quality of their events and the satisfaction of the participants in order to strengthen the events over time. This work investigates a holistic model of relationships between global quality, perceived value, general satisfaction, and future intentions under the moderating effect of emotions of the participants in a sporting event. The results undoubtedly serve to guide managers to plan this specific type of service to utilize such strategies.

The results of the analysis reveal that good indicators of adjustment, reliability, and the validity of measurements empirically support that the measurement model is adequate to a considerable extent. Each construct of the model is autonomous, different from the rest, and representative of the dimension that it wants to indicate. So, these results provide more evidence to the usefulness of the model proposed by Angosto-Sánchez et al. (2016b) for sports modalities of the triathlon federation. The structural model identifies which items and dimensions are key for the participants in the duathlon, when determining the perceived quality and global quality of the event. This confirms the convenience of establishing the implicit dimensions in a specific scenario or event (Taylor et al., 1993; Theodorakis et al., 2015) since they affect the model, and the opportunity to consider sub-dimensions to improve the appreciation of global quality (Clemes et al., 2011).

The results for this specific event highlight the staff and the specific elements of a duathlon. In addition, these reinforce the general idea in sports management of the importance of staff (Kelley and Turley, 2001; Theodorakis et al., 2001; Hightower et al., 2002; Bodet, 2006; Kim and Severt, 2011; Ko et al., 2011; Crespo-Hervás et al., 2012) and the specific environment (Westerbeek and Shilbury, 2003). It is also confirmed that the general quality dimensions have a direct positive effect on the general satisfaction of the event, results that are similar to other authors (Zeithaml et al., 1996; Brady and Cronin, 2001; Nuviala et al., 2015). However, the influence of global quality on perceived value is not observed, unlike other studies that do support this relationship (Theodorakis et al., 2001; Clemes et al., 2011; Calabuig-Moreno et al., 2015, 2016a). This lack of relationship suggests that the participants in this event give more importance to quality than to the associated costs of attending. The study also does not support the relationship between value and general satisfaction, in contrast to the study of Murray and Howat (2002), Clemes et al. (2011), or that of Calabuig-Moreno et al. (2016a). The results also give more importance to the emotional aspects related to satisfaction than to value. The SEM analysis discovers that general satisfaction is a good predictor of future intentions, as well as, indirectly, the staff, duathlon aspects, and global quality, but not the perceived value, as other recognized studies have said (Cronin et al., 2000; Hightower et al., 2002; Calabuig-Moreno et al., 2015, 2016a,b). Therefore, the research reveals that emotional involvement associated with sports participation and affective aspects that condition satisfaction influences an individual’s intention to attend a similar event in the future.

The SEM analysis shows that emotions (pleasure and arousal) alone or indirectly are not explanatory of the future intentions of the participants. However, with the qualitative study fsQCA, the combinatorial effects have been estimated and sufficient combinations of conditions have been identified that do explain the incidence of emotions in the final effect studied. Thus, the application of fsQCA allows us to affirm that emotions, although by themselves are not a predictor of future intentions, in combination with other variables are very important. In fact, the three combinations of sufficiency conditions obtained bear in mind the two emotions considered in this work (pleasure and arousal).

We conclude that it has been appropriate to carry out an analysis of the relationships between quality, satisfaction, perceived value, future intentions, and emotions in participants of the European Duathlon Championship, as it has provided knowledge to managers of similar duathlon or triathlon events. This study suggests that in the management of sporting events, the emotions of the participants must be considered as precursors to behavioral intentions. Furthermore, the introduction of fuzzy logic has increased the explanatory value of the model. So, it seems convenient to combine the linear models and the fsQCA model to improve analyzes in the future.

The main limitation of this study is the low sample size, since a larger size would have allowed for creating more consistent and reliable fuzzy numbers. Furthermore, this specific type of event is not representative of others, making it difficult to generalize the results to all kinds of sporting events. However, we consider that the results are a useful representation of the phenomenon studied, in view of the limited number of research in the literature that address the phenomenon from the perspective of the participants. We suggest applying this study to participants from similar events and different contexts. We propose that how the outcome of the competition affects individual’s future intentions should be analyzed and, moreover, to include the study of the influence of sex, nationality, or the modality of competition into the evaluations of the dimensions. These issues will be useful to guide managers toward the development of efficient strategies focused on different segments of participants. Although this exceeds the limits of this work, it generates appealing futures lines of research.

During the completion of this investigation, many people have been living through lockdowns as a result of the Covid-19 pandemic. This situation will undoubtedly affect the holding of future events and the relationship between the agents involved. Therefore, it is hypothesized that there will be a need to design security protocols that can determine an individual’s decision to attend an event or not. Therefore, new highly relevant lines of research have been generated.

## Implications for Management

In accordance with these results, managers are encouraged to focus on guiding staff functions toward participant satisfaction, focusing especially on amateur participants. It is suggested, for better efficiency, to pay special care to the design process and control of the technical aspects of the race. In addition, it is advised to develop strategies to increase pleasure and arousal during the moments before the celebration, during the trial, and at the end of it. Therefore, the main advice for managers would be to focus their efforts on offering high levels of perceived quality and satisfaction to participants, especially amateur participants.

## Data Availability Statement

The raw data supporting the conclusions of this article will be made available by the authors, without undue reservation, to any qualified researcher.

## Ethics Statement

Ethical review and approval was not required for the study on human participants in accordance with the local legislation and institutional requirements. Written informed consent to participate in this study was provided by the all participants.

## Author Contributions

AM-G put forward a theoretical idea and collected the data. CS-P and CM-C analyzed the data and wrote it into the article. MG-T revised the theoretical framework and organized the work. All authors listed have made a substantial, direct and intellectual contribution to the work, and approved it for publication.

## Conflict of Interest

The authors declare that the research was conducted in the absence of any commercial or financial relationships that could be construed as a potential conflict of interest.

## Footnotes

1 http://www.socsci.uci.edu/~cragin/fsQCA/software.shtml
